# Primary Hydatid Cyst of Neck Misdiagnosed as Lipoma: A Rare Case Report From Nepal

**DOI:** 10.1002/ccr3.71108

**Published:** 2025-10-01

**Authors:** Khusbu Kumari, Ishwor Thapaliya, Sumesh Singh, Susmin Karki, Simin Kunwar

**Affiliations:** ^1^ Tribhuvan University, Institute of Medicine Maharajgunj Nepal

**Keywords:** echinococcus, histopathology, Hydatidosis, neck mass, surgery, ultrasonography

## Abstract

Hydatid disease, caused by Echinococcus tapeworm infection, presents a significant health concern, particularly in low and middle‐income countries. This zoonotic disease predominantly affects the liver and lungs but can occur in various locations throughout the body. Here, we report a rare case of a 10‐year‐old girl with a hydatid cyst in the nape of her neck. Initially misdiagnosed as a lipoma on clinical examination, ultrasonography revealed the cystic nature of the mass, and histopathological examination done after surgery confirmed the diagnosis. This case highlights the importance of considering hydatid cysts in the differential diagnosis of neck masses among pediatric patients in endemic regions to ensure timely diagnosis and appropriate management.

## 1 Introduction

A zoonotic disease called Hydatidosis results from Echinococcus tapeworm infection, particularly by the meta‐cestode stage of *E. granulosus* , and less commonly E. multilocularis [1, 2]. It is a common parasitic infection in low and middle‐income countries [3, 4]. Echinococcus has a life cycle involving dogs as definitive hosts and humans as accidental intermediate hosts, typically infected after ingestion of food and water contaminated with parasite eggs or by contact with an infested dog [5]. This leads to cyst formation primarily in the liver (75%) and lungs (15%) [1, 4]. However, cysts can occur anywhere in the body after eggs have passed through two pulmonary and hepatic filters, as reported by numerous studies [6, 7, 8]. It may present in uncommon locations and can be mistaken for other causes of cysts. The isolated occurrence of a cyst in the soft tissue of the neck is rare even in endemic regions, comprising only 1% of all human echinococcosis sites [4, 9]. Thus, accurate pre‐operative diagnosis is important for effective management [3].

In the medical literature, only eight cases of hydatid cyst in the nape of the neck have been reported to date, with no instances documented in Nepal. This marks the first case of a hydatid cyst in a rare location, highlighting the importance of considering them in differential diagnoses of cervical masses.

## 2 Case History/ Examination

A 10‐year‐old girl with no known comorbid conditions presented to our Pediatric surgery outpatient department complaining of painless swelling on the nape of her neck, progressively growing over the past year. There was no prior history of fever, neck muscle spasms, breathing difficulty, voice changes, ENT bleeding, or hearing issues. She had no significant medical, surgical, or family history.

Physical examination revealed a soft, mobile mass measuring 6 × 5 cm with slight tenderness upon palpation. The overlying skin was unaffected, with no pulsations observed. The systemic examination revealed no significant findings.

## 3 Differential Diagnoses

The initial differential diagnosis included lipoma, due to the soft, mobile nature of the mass and lack of systemic symptoms. Cystic hygroma and lymphadenopathy were also considered, along with a parasitic cyst given the eosinophilia.

## 4 Investigations

Laboratory test results reveal eosinophilia (12.8%) and raised prothrombin time (16 s). Other laboratory parameters were within normal range as illustrated in Table 1. Ultrasound revealed a well‐defined cystic structure measuring 4 × 4.6 × 3.3 cm with posterior acoustic shadowing in the nape of the neck. No echotexture or vascularity was observed. Although an MRI was planned, due to financial constraints it could not be done.

**TABLE 1:** Laboratory findings of the patient at the time of presentation.

Laboratory parameters	Results	Units	Reference value
Complete blood cell count			
Hemoglobin	12.9	g%	12–16
Hematocrit	36	%	36–48
Total leukocyte count (TLC)	6900	/mm^3^	4000–11,000
Platelet count	355,000	/mm^3^	150,000–450,000
Differential count			
Lymphocytes	42.7	%	25–45
Neutrophils	39.4	%	45–75
Monocytes	5.1	%	2–10
Eosinophils	12.8	%	1–6
Coagulation profile			
Prothrombin time (PT)	16	s	10–13
International normalized ratio (INR)	1.16	—	0.8–1.2
Biochemical tests			
Sodium	136	meq/L	135–145
Potassium	4.7	meq/L	3.5–5.5
Random blood sugar	4.7	mmol/L	3.8–7.8
Blood urea	3.6	mmol/L	1.8–6.4
Serum creatinine	41	μmol/L	45–105
Urine analysis			
Leucocytes	0–1	/hpf	0–5
Epithelial cells	0–1	/hpf	0–4
Serological analysis			
HCV	Negative		
HIV	Nonreactive		
HBsAg	Negative		

Abbreviation: hpf, high power field.

## 5 Treatment

**FIGURE 1:**
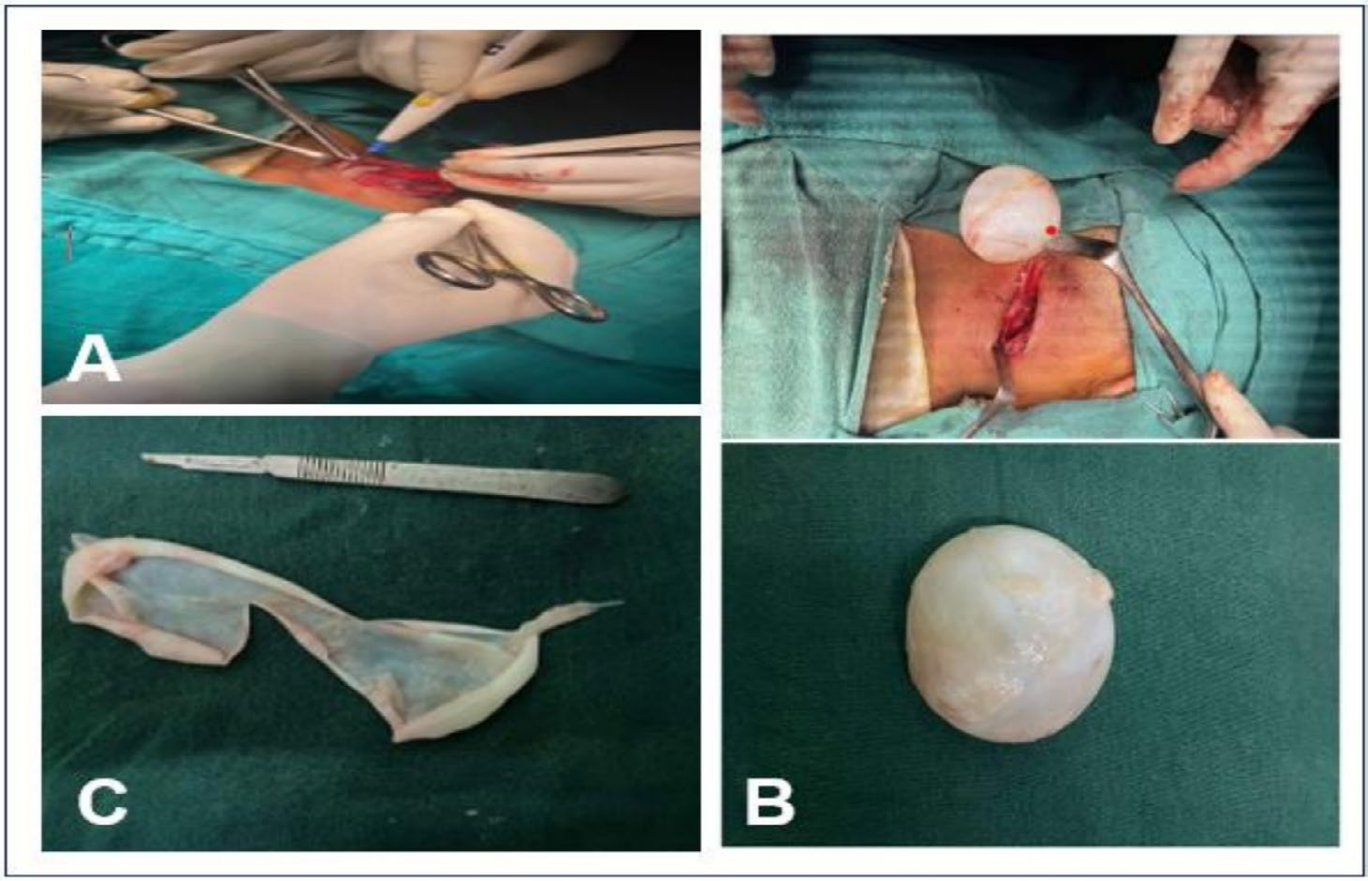
Intraoperative images and specimen after resection, (A) Surgical excision for removal of hydatid cyst; (B) Gross image of hydatid cyst measuring about 6.5 × 6.5 × 4 cm in size; (C) Hydatid cyst on cut sectioning.

**FIGURE 2:**
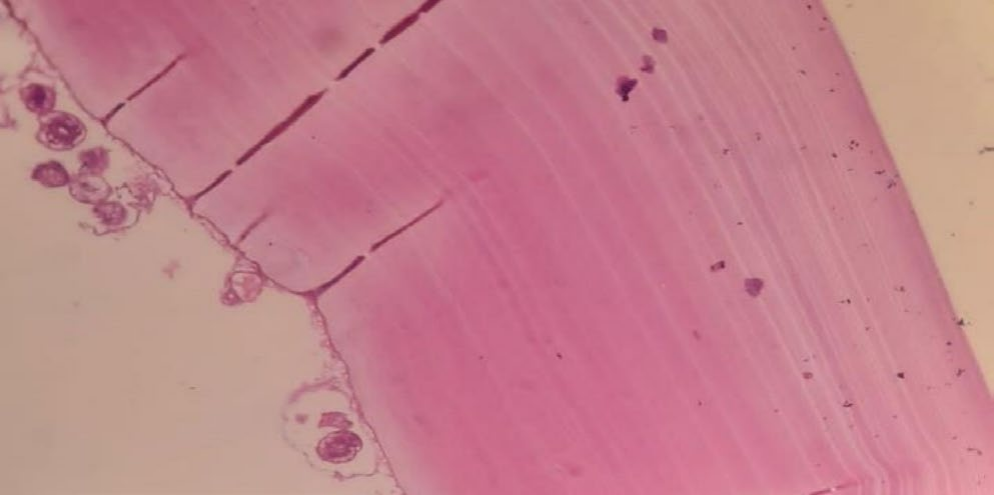
Histopathological image of hydatid cyst showing granulation tissue and mixed inflammatory cells stained with hematoxylin and eosin stain (H &E stain); 10× original magnification.

Surgery was scheduled for the patient. Intraoperatively, a thick‐walled single cyst measuring about 6.5 × 6.5 × 4 cm was observed, without invasion into surrounding structures as shown in Figure 1a,b. On cut sectioning, the cyst was unilocular, filled with a white gelatinous membrane as shown in Figure 1c. Histopathological examination revealed an avascular, acellular, lamellated layer with a fibrous wall, showing granulation tissue and mixed inflammatory cells as illustrated in Figure 2. Proto‐scoliosis (ovoid daughter cysts) within parasitic hooklets was also noted.

## 6 Outcome and Follow Up

Postoperative period was uneventful and the patient was kept on albendazole 800 mg/day for 4 weeks. A follow‐up examination after six months showed no complications.

## 7 Discussion

Hydatid cysts have been reported in various locations such as the axilla, spleen, heart, bones, muscles, kidney, and cranium with soft tissue involvement in less than 3% of cases [10]. In the cervical region, they are extremely rare, constituting only 0.75% of all hydatid cysts [11]. All age groups can be affected by this disease with a sex ratio of 1:1, but studies have shown a notable predominance among the pediatric population [4]. The estimated prevalence rate of disease ranges from 0.6 to 1.2 per 100,000 individuals. It accounts for approximately 1% of all surgical admissions [3]. Neck involvement is believed to result from the systemic circulation of Echinococcus embryos through the lymphatic system [12].

The majority of neck hydatid cysts are asymptomatic, with variable signs and symptoms depending on location, size, and pressure effect of cysts [13, 14]. It can grow anywhere between 1 and 5 cm per year [15, 16]. Neck hydatid cysts are more often discovered incidentally as painless, slowly growing masses [4, 12, 13, 14]. In some cases, compression of nearby structures may lead to symptoms like dyspnea or dysphagia [4, 14, 17]. The absence of specific clinical signs often leads to misdiagnosis of hydatid disease, with conditions such as cystic lymphangioma, cold abscess, chronic hematoma, bronchial cleft cyst, thyroglossal duct cyst, epidermal cyst, and lipoma [3].

Patients should undergo comprehensive systemic evaluation, as 20%–30% of cases involve multiple systems, particularly the liver and lungs [14, 18]. However, our patient showed no evidence of hydatid cysts elsewhere in the body apart from the neck. This isolated occurrence, without involvement in other sites, is unusual and can lead to misdiagnosis, as in our case, initially diagnosed as lipoma but changed intraoperatively. Past medical history, family history, the patient's occupation, history of dog bite, and residence can provide clues to consider a hydatid cyst in the differential diagnosis. However, preoperative diagnosis may be overlooked without suspected or demonstrative radiologic findings [13].

The diagnosis of hydatid disease is greatly aided by ultrasound (USG), CT, and magnetic resonance imaging (MRI) [14]. Although MRI offers better anatomical and structural assessment with signs of compression [4], it wasn't performed in our case due to financial constraints. In resource‐limited settings like Nepal, where many peripheral health centers lack resources, ultrasound (USG) remains the preferred and cost‐effective method for diagnosing hydatid disease. It is estimated to have a sensitivity and specificity of 90%–95% [12]. It clearly reveals hydatid sands, floating membranes, daughter cysts, and vesicles in purely cystic lesions, aiding physicians in achieving early and accurate diagnosis [13]. Serology can diagnose hydatid disease in 80%–90% of cases, but negative results don't exclude the diagnosis [4, 12]. The application of Fine‐needle aspiration cytology (FNAC) remains controversial due to the risk of fatal anaphylactic reactions from cyst rupture [13]. Biopsy should be avoided prior to surgery if a cystic mass is identified on ultrasonography performed preoperatively [4].

Surgical modalities like cystectomy and capitonnage remain the gold standard surgical approaches for complete removal of hydatid cysts [13, 14]. During surgery, the cyst was removed carefully in our case to prevent spillage of cystic contents. Spillage may lead to multiple hydatosis in the neck in the future [13]. The observation of whitish, clear cystic fluid with a germinative layer during gross examination may be adequate for intraoperative diagnosis of the hydatid cyst [11]. Histopathological examination confirms the diagnosis by identifying a trilaminar cyst wall consisting of an outer acellular membrane and an inner nucleated germinal membrane, sometimes with scolexes as observed in our case [4]. Pre‐ and post‐operative administration of antiparasitic drugs can reduce the risk of contamination and recurrence [11, 19].

## 8 Conclusion

Clinicians should consider hydatid cyst as a differential diagnosis, especially in endemic regions and among pediatric patients where neck masses are common clinical presentations. A misdiagnosis of hydatid cyst as lipoma can delay appropriate management, potentially leading to serious implications for the patient's life. Careful clinical examination, detailed history, and at least ultrasonography prior to treatment are crucial for timely diagnosis and management of such atypical presentations in resource‐limited settings.

## Author Contributions

**Khusbu Kumari:** writing – original draft, writing – review and editing. **Ishwor Thapaliya:** writing – original draft, writing – review and editing. **Sumesh Singh:** writing – review and editing. **Susmin Karki:** writing – review and editing. **Simin Kunwar:** writing – review and editing.

## Ethics Statement

Since this is a case report, our Institutional Review Board has waived the requirement for ethical approval.

## Consent

A written informed consent was obtained from the patient to publish this report in accordance with the journal's patient consent policy.

## Conflicts of Interest

The authors declare no conflicts of interest.

## Data Availability

All of the data generated during this study can be accessed through direct communication with the corresponding author and with the approval of the entire research team.
